# Integrated Modules Analysis to Explore the Molecular Mechanisms of Phlegm-Stasis Cementation Syndrome with Ischemic Heart Disease

**DOI:** 10.3389/fphys.2018.00007

**Published:** 2018-01-22

**Authors:** Wei-Ming Xu, Kuo Yang, Li-Jie Jiang, Jing-Qing Hu, Xue-Zhong Zhou

**Affiliations:** ^1^Research Centre for Disease and Syndrome, Institute of Basic Theory for Traditional Chinese Medicine, China Academy of Chinese Medicine Sciences, Beijing, China; ^2^School of Computer and Information Technology and Beijing Key Lab of Traffic Data Analysis and Mining, Beijing Jiaotong University, Beijing, China; ^3^Data Center of Traditional Chinese Medicine, China Academy of Chinese Medical Sciences, Beijing, China

**Keywords:** ischemic heart disease, phlegm-stasis cementation syndrome, network medicine, disease module, systems biology

## Abstract

**Background:** Ischemic heart disease (IHD) has been the leading cause of death for several decades globally, IHD patients usually hold the symptoms of phlegm-stasis cementation syndrome (PSCS) as significant complications. However, the underlying molecular mechanisms of PSCS complicated with IHD have not yet been fully elucidated.

**Materials and Methods:** Network medicine methods were utilized to elucidate the underlying molecular mechanisms of IHD phenotypes. Firstly, high-quality IHD-associated genes from both human curated disease-gene association database and biomedical literatures were integrated. Secondly, the IHD disease modules were obtained by dissecting the protein-protein interaction (PPI) topological modules in the String V9.1 database and the mapping of IHD-associated genes to the PPI topological modules. After that, molecular functional analyses (e.g., Gene Ontology and pathway enrichment analyses) for these IHD disease modules were conducted. Finally, the PSCS syndrome modules were identified by mapping the PSCS related symptom-genes to the IHD disease modules, which were further validated by both pharmacological and physiological evidences derived from published literatures.

**Results:** The total of 1,056 high-quality IHD-associated genes were integrated and evaluated. In addition, eight IHD disease modules (the PPI sub-networks significantly relevant to IHD) were identified, in which two disease modules were relevant to PSCS syndrome (i.e., two PSCS syndrome modules). These two modules had enriched pathways on Toll-like receptor signaling pathway (hsa04620) and Renin-angiotensin system (hsa04614), with the molecular functions of angiotensin maturation (GO:0002003) and response to bacterium (GO:0009617), which had been validated by classical Chinese herbal formulas-related targets, IHD-related drug targets, and the phenotype features derived from human phenotype ontology (HPO) and published biomedical literatures.

**Conclusion:** A network medicine-based approach was proposed to identify the underlying molecular modules of PSCS complicated with IHD, which could be used for interpreting the pharmacological mechanisms of well-established Chinese herbal formulas (*e.g., Tao Hong Si Wu Tang, Dan Shen Yin, Hunag Lian Wen Dan Tang and Gua Lou Xie Bai Ban Xia Tang*). In addition, these results delivered novel understandings of the molecular network mechanisms of IHD phenotype subtypes with PSCS complications, which would be both insightful for IHD precision medicine and the integration of disease and TCM syndrome diagnoses.

## Background

Ischemic heart disease (IHD), caused by the atherosclerosis of coronary vessels, is also called coronary atherosclerosis heart disease. IHD has remained the leading cause of death during the past several decades, according to a 2017 WHO report (World Health Organization, 2017). In recent years, many progresses have been made in identifying the underlying mechanisms of IHD in terms of genetic architecture (Tikkanen and Helio, 1994; Samani, 1998; Ma and Liew, 2003; Wang, 2005; Liu H. et al., 2011; Wang et al., 2011; Hua et al., 2014; Roberts, 2014; Ozaki and Tanaka, 2016). For example, the CADgene database, a comprehensive database for Coronary Heart Disease (CAD) associated genes, have been curated by collecting relevant literatures from PubMed (Liu H. et al., 2011). However, as a complex disease, IHD incorporates several disease subtypes, including ariant/unstable angina pectoris, myocardial infarction, acute coronary syndrome and cardiac sudden death (Chinese Minister of Health, 2010). These subtypes represent different stages of disease development, and additional genetic profiles (e.g., genetic interaction network) should be investigated to better understand the molecular mechanisms of IHD.

Syndrome (also called ***Zheng***) is the basic diagnostic unit and a key concept in Traditional Chinese Medicine (TCM) (Gu, 1956). Syndromes could be regarded as phenotype subtypes of diseases to a certain degree. According to TCM theory, a syndrome is a combination of clinical manifestations that commonly occur together, thus implying the existence of a particular disease condition (Wang and Xu, 2014; Zhao Y. F. et al., 2014). One syndrome always consists of a characteristic profile of all clinical manifestations, including signs, symptoms, tongue appearances, and pulse feelings (briefly called symptoms in the following text), in a patient these are identifiable to TCM practitioners. Phlegm syndrome (abbreviated as PS) and blood-stasis syndrome (abbreviated as BS) are both among the most common syndromes in IHD patients in China (Mao et al., 2011; Ren et al., 2012; Li et al., 2014). For example, a comprehensive retrieval of CAD syndromes published in the China National Knowledge Infrastructure (CNKI) database and VIP Information Network databases from 1970 to 2010 indicated that the main syndromes were primarily associated with BS and PS, and the incidence of both had increased in recent years (Mao et al., 2011). Another clinical epidemiological investigation on current syndrome characteristics of 8,129 IHD patients indicated that BS appeared in 77.89% of IHD patients and PS appeared in 43.97% of IHD patients (Bi et al., 2017). Phlegm-stasis cementation syndrome (abbreviated as PSCS), which represents both PS and BS in IHD patients, occurred in 24.33~26.22% of IHD patients and was significant complication with IHD (Bi et al., 2013, 2017).

Following syndrome differentiation, TCM physicians usually utilize herb prescriptions for disease treatments. Several classical formulas have been used for thousands of years to treat BS and PS complicated with IHD. For example, *Huang Lian Wen Dan Decoction*, a formula to treat PS with IHD in TCM clinical practice, can ameliorate the clinical symptoms of stable angina pectoris, reduce the incidence of angina and delay the progression of atherosclerosis (Gong, 2012; Yan et al., 2015). *Gua Lou Xie Bai Ban Xia Decoction*, a classical formula created by the medical sage Zhang Zhong-jing over 2000 years ago, exerts a protective effect on the ischemic rabbit myocardium, and the underlying mechanisms may involve the inhibition of nitric oxide synthase (NOS) activity and are duction in the excessive production of nitric oxide (NO) (Zhou et al., 2010; Zhang et al., 2011). *Dan Shen Yin Decoction* and *Tao Hong Si Wu Decoction* are both well-known decoctions used to treat BS with IHD (Yan et al., 2012; Yin et al., 2013; Liu et al., 2015).

Network medicine (Barabasi et al., 2011), particularly that involving disease modules, is a promising approach to investigate the network mechanisms of complex diseases (Goh et al., 2007; Menche et al., 2015), particularly for disease subtypes (Wang et al., 2017), disease phenotypes and disease-disease associations (Barabasi et al., 2011; Chen and Butte, 2013; Wang et al., 2017). Transcriptomics, metabolomics, proteomics, and other omics technologies have the potential to provide new insights into complex disease pathogenesis and heterogeneity, especially if they are applied within a network biology framework (Silverman and Loscalzo, 2012). Recent studies have tried to investigate the association of one symptom with one syndrome [such as quantitative facial color features with cold pattern (Mun et al., 2017)] or understanding the syndrome from the view of genotypes-phenotypes interactions (Chung, 2014; Fraser et al., 2015). And the main strategies include: (1) conducting the metabonomic and proteomic research (Shi et al., 2014b; Zou et al., 2014; Jiang et al., 2015; Sun et al., 2015); (2) constructing MiRNA-target network (Liao et al., 2016; Liu et al., 2017); (3) integrating the classical formulas or herb pair (Chen et al., 2016; Xu et al., 2016; Zhou et al., 2016; Yue et al., 2017); (4) analyzing compound–nature pairs from TCM via chemical space visualizations (Liang et al., 2013; Fu et al., 2017); (5) using compound-target-disease associations to reconstruct the biologically-meaningful networks based on systems pharmacology (Zhou and Wang, 2014). Moreover, in recent years, a growing number of studies have focused on the biological mechanisms underlying BS with IHD (Mao et al., 2004; Liu Y. et al., 2011; Chen, 2012; Hao et al., 2013; Huang et al., 2013; Su et al., 2013; Wang and Yu, 2013; Li et al., 2015), PS with IHD (Wang et al., 2009; Zhao, 2009; Fang et al., 2011; Kong et al., 2014) and PSCS with IHD (Zhang et al., 1995, 1999; Liu et al., 2008; Bai and Song, 2012; Lin et al., 2014; Zhao L. et al., 2014; Ren et al., 2015). However, the molecular mechanisms of PSCS with IHD have not been fully elucidated clearly and have not yet been investigated from a network medicine perspective (Hopkins, 2007; Li and Zhang, 2013), in particular the symptoms or cluster of symptoms (corresponding to specific syndromes) have been always ignored to be explored in system biology (Zhou et al., 2014a,b), although symptoms were the most common and focused phenotypes in TCM (Chung, 2014).

**Figure 1:**
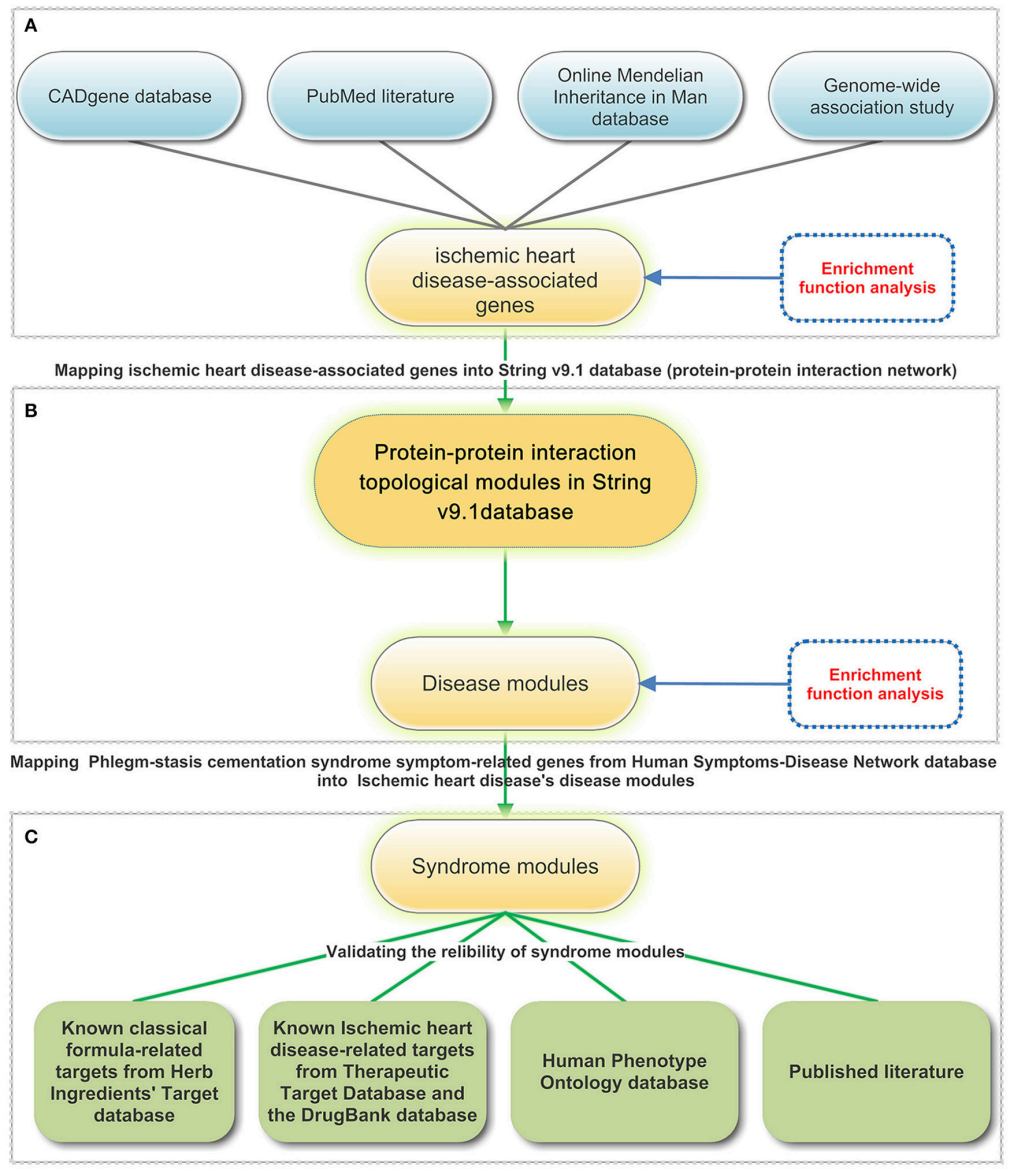
The major steps of syndrome module development. To obtain the most reliable results, the following steps were taken: **(A)** Ischemic heart disease (IHD)-associated genes were integrated from three different sources: the CADgene database, PubMed literatures, and the Online Mendelian Inheritance in Man (OMIM) database and Genome-wide association study (GWAS). Gene Ontology (GO) and pathway enrichment functional analysis were conducted on ischemic heart disease—associated genes set. **(B)** Following the detection of protein-protein interaction (PPI) topological modules in the String V9.1 database, ischemic heart disease—associated genes were mapped into the protein-protein interaction (PPI) topological modules to obtain disease modules. Gene Ontology (GO) and pathway enrichment functional analyses were carried out for the disease modules. **(C)** Syndrome modules were identified by mapping phlegm-stasis cementation syndrome (PSCS) symptom-related genes from Human Symptoms-Disease Network (HSDN) into disease modules. Syndrome reliability was validated with known classical formula-related targets from the Herb Ingredients' Targets (HIT), known ischemic heart disease-related targets from the Therapeutic Target Database (TTD and DrugBank databases), the Human Phenotype Ontology (HPO) and published literatures.

In this study, we proposed a network medicine-based approach to identify the underlying molecular modules of PSCS complicated with IHD. We firstly identified protein-protein interaction (PPI) topical modules that were closely related to PSCS with IHD and explored the molecular mechanisms of PSCS with IHD via integrated modules analysis. PSCS with IHD associated syndrome modules were detected by integrating symptom-gene relationships based on the identification of IHD disease modules. Under the theoretical guideline of correspondence between prescription and syndrome (Gao et al., 2012; Lu et al., 2014), *Dan Shen Yin Decoction* and *Tao Hong Si Wu Decoction* were both classical formulas to treat BS with IHD not for PS with IHD, *Gua Lou Xie Bai Ban Xia Decoction* and *Huang Lian Wen Dan Decoction* were both classical formulas to treat PS with IHD not for BS with IHD. When Chinese Medicine treated the PSCS, treatments for PS and BS with IHD should been used synchronously. Targets of the four classical formulas and known IHD-related targets were used to validate the reliability of the syndrome modules because network pharmacology had become an important approach in TCM to understand the underlying mechanisms of syndromes (Li and Zhang, 2013; Li et al., 2013; Pei et al., 2013; Zhang et al., 2013, 2017; Yue et al., 2017). The Human Phenotype Ontology (HPO) and published literatures were also examined to gather supplementary evidences. Ultimately, we identified two modules that were closely associated with PSCS with IHD and explored their molecular network mechanisms by performing molecular functional analyses. The main steps in our work were as shown in Figure 1.

## Materials and methods

### Curation of IHD-associated genes

We included all subtypes of IHD as MeSH terms and integrated three different data sources: the CADgene database (Liu H. et al., 2011), PubMed and Disease-Connect [including Online Mendelian Inheritance in Man database (OMIM) and Genome-wide association study (GWAS)] (Liu et al., 2014). We incorporated all genes from the CADgene database as IHD-associated genes. For PubMed literatures, we adopted systematic steps to manually curate disease-gene associations from published records. First, we manually selected MeSH terms related to IHD subtypes from the MeSH terminology (https://www.nlm.nih.gov/mesh/2014). Then, we manually searched the PubMed database using each MeSH term to obtain records related to IHD and its human genes. Next, we filtered the records by querying the PubMed database using the full names or symbols of genes from the National Center for Biotechnology Information (NCBI) gene database. Finally, we identified IHD MeSH terms and their associated genes in terms of their co-occurrence in PubMed records. Because CADgene and PubMed records may contain repetitive associations, we calculated the overlap between the CADgene and PubMed records. After excluding these records, we manually checked the remaining associations between genes and disease subtypes. One association might have multiple related biographical records, and we considered the association to be true if at least one record indicated the relationship between the query gene and a disease subtype existed. Another data source for disease-gene associations was the Disease-Connect database (Liu et al., 2014) (Accessed on Feb 5, 2015), from which we extracted the OMIM and GWAS subset.

### Identification of disease modules

Community structures were widely distributed within complex networks. Each community comprised nodes that densely connected its members and were sparsely connected with the nodes in other modules (Dittrich et al., 2008). Firstly, we obtained a reliable PPI network that included 15,524 nodes (proteins) and 218,409 edges (protein-protein associations) by filtering high-quality protein-protein associations (weight of edges > 700) from the String V9.1 database (Szklarczyk et al., 2011; Franceschini et al., 2013). Then, we applied a widely used algorithm called BGLL (Subelj and Bajec, 2011) to obtain PPI topological modules. The PPI network was partitioned into 314 modules. Modules containing at least one IHD-associated gene were regarded as potential disease modules. By calculating p-values (hypergeometric distribution) between IHD-associated genes and potential disease modules, all enriched modules (*p*-value < 0.01) were considered disease modules.

### Identification of syndrome modules

By mapping PSCS symptom-related genes into potential disease modules and calculating p-values (hypergeometric distribution) between PSCS-associated genes and potential disease modules, all enriched modules (*p*-value < 0.01) that simultaneously overlapped with disease modules were deemed syndrome modules. PSCS symptom-gene relationships were obtained from the Human Symptoms-Disease Network (HSDN) in our previous study (Zhou et al., 2014b). All symptoms consistent with PSCS with IHD were derived from the diagnostic criteria for PSCS with IHD in our previous study (Hu et al., 2016). We combined the diagnostic criteria for BS with IHD (Fu et al., 2012) and PS with IHD (Hu et al., 2016) (China Association of Traditional Chinese Medicine, 2017) to establish the diagnostic criteria for PSCS with IHD. In the diagnostic criteria for PSCS with IHD, PSCS with IHD involved 25 symptoms in total: chest tightness/chest heaviness, heaviness in limbs, sticky mouth, sticky stool, abdominal distension, dark cloudy complexion, decreased appetite/appetite absent, petechiae /cracks, dark purple lips/gums, abnormal sublingual veins, squamous and dry skin, darkish complexion, ecchymosis, body weight, morbidobesity, cyanosis, lethargy, chest pain, dark purple tongue, fat tooth-imprinted tongue, greasy coating, white tongue coating, soft pulse, slippery pulse, and uneven pulse. The 25 Chinese symptom terms were manually translated into terms with English version by TCM researcher, and these terms were automatically matched against the “symptom” and “disease” terms in Medical Subject Headings (MeSH) containing a large number of standard medical terminologies (https://www.nlm.nih.gov/mesh/meshhome.html#, accessed on Oct.30, 2014) and the side effect-related terms in the SIDER database (Kuhn et al., 2016).

### Reliability validation

Four different aspects of information were used to validate the reliability of the syndrome modules: known classical formula-related targets from the Herb Ingredients' Targets (HIT) (Ye et al., 2011), known IHD-related targets, the HPO (Kohler et al., 2017) and the published literatures. Effective ingredients and their corresponding targets from herbs contained in *Huang Lian Wen Dan Decoction, Gua Lou Xie Bai BanXia Decoction, Dan Shen Yin Decoction* and *Tao Hong Si Wu Decoction* were integrated from the HIT (accessed on Apr.12, 2015; Ye et al., 2011). As we did not predict the exact number of targets in these formulas, one disease modules may include BS's classical formula-related targets and PS's classical formula-related targets at the same time, the difference of occurrence probability would be affected by the unequal number of the herbs consisted of classical formulas. In order to avoid the difference, we added one herbs Rhizoma Acori Talarinowii (ShiChangPu 石菖蒲 in Chinese) as an herb utilized to treat PS with IHD because this herb was widely used to eliminate phlegm syndrome according to TCM theory and clinical practice (Gao et al., 2015). Thus, we ensured the number of herbs used to treat PS with IHD was equal to the number of herbs used to treat BS with IHD. Then, these known classical formula-related targets were mapped into disease modules. Known IHD-related targets were obtained from the Therapeutic Target Database (TTD) (accessed on Apr.10, 2015) (Zhu et al., 2012) and the DrugBank database (accessed on Apr.10, 2015) (Wishart et al., 2008; Law et al., 2014) under the assumption that modules containing known IHD-related targets were more accurate and reliable than modules that did not contain any IHD-related target. In the TTD, IHD-related targets were identified using 20-25 corresponding International Classification of Diseases (ICD) disease names. In the DrugBank database, disease names were used as keywords to search for IHD-related targets. The HPO, independent of the HSDN database, was a computational representation of a knowledge domain based upon a controlled, standardized vocabulary to describe entities and the semantic relationships between them (Kohler et al., 2017). So HPO was used to validate symptom-gene relationships in the syndrome modules. Published literatures were also collected to validate the reliability of syndrome modules.

### Enrichment analyses

To validate the reliability of the integrated results and explore the molecular mechanisms of disease modules (syndrome modules belonged to disease modules), gene ontology (GO), and pathway enrichment analyses were carried out on IHD-associated gene set and disease modules. There are many online analysis platforms and tools to conduct GO enrichment analysis and Kyoto Encyclopedia of Genes and Genomes (KEGG)^1^ pathway analysis (Khatri and Draghici, 2005), in our study, we used the KOBAS 2.0 database (Xie et al., 2011). Gene Ontology database and KEGG PATHWAY database were chosen for analysis in the KOBAS 2.0 settings. By calculating the hypergeometric distribution relationship, we obtained statistically significant GO terms and pathways. We performed Bonferroni corrections to control the false-positive rate in the analysis, and finally filtered the disease modules by CPVs (corrected *p*-values), which resulted in significant GO terms and pathways (CPV < 0.01).

## Results

### IHD-associated genes

**Figure 2:**
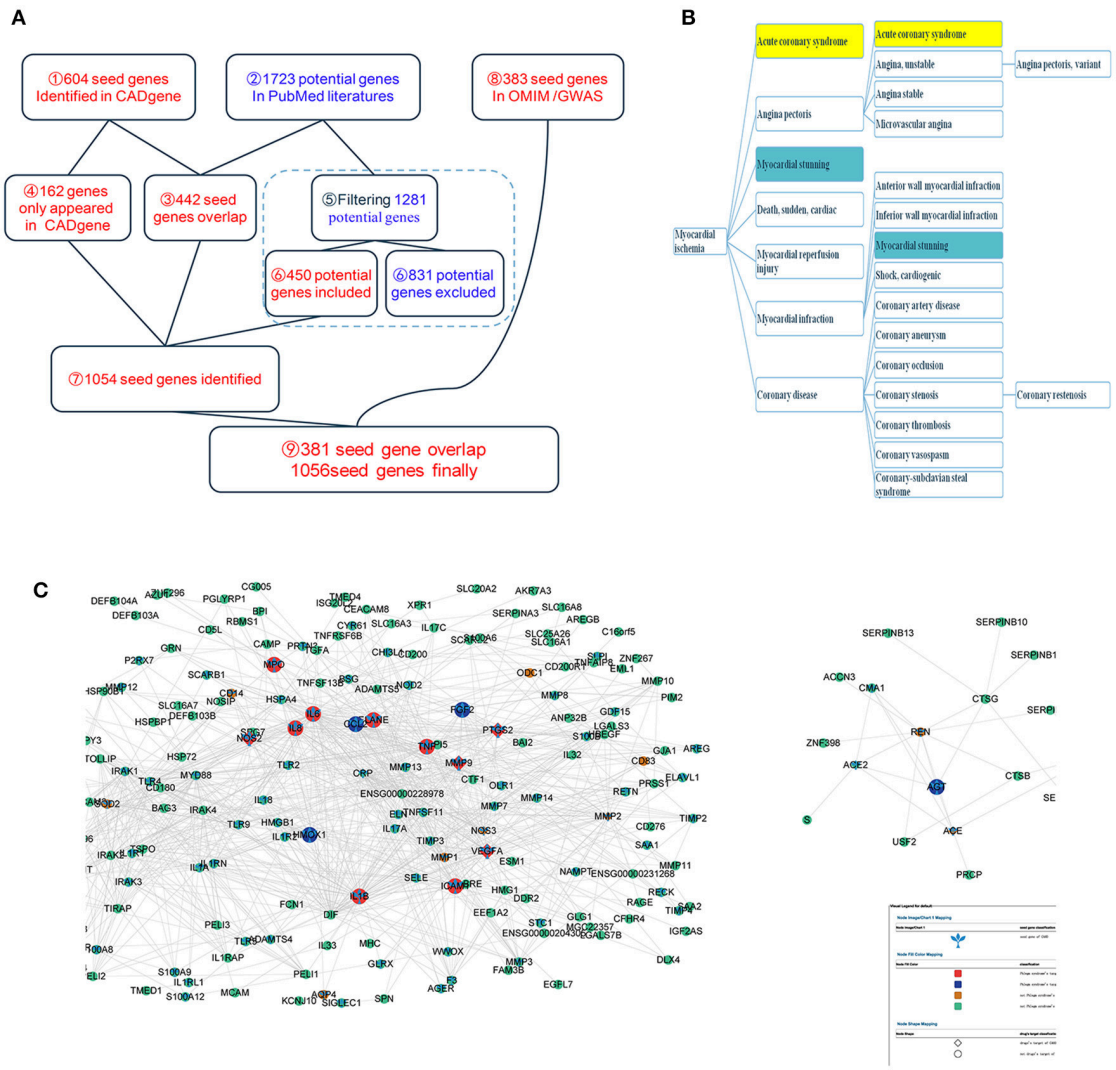
The main results of the study. **(A)** The steps and process how ischemic heart disease associated genes formed. **(B)** The upper and down relationships between the MeSH headings of ischemic heart disease (IHD). **(C)** Modules 195 and 95 representing phlegm-stasis cementation syndrome (PSCS) with ischemic heart disease (IHD) (syndrome modules).

As shown in Figure 2A and Table S1–4, a total of 604 IHD-associated genes were obtained from the CADgene database (accessed on Oct.30, 2014, see Table S1). As shown in Table 1 and Figure 2B, MeSH term headings referring to IHD and its subtypes (up-down relationships between subtypes also included) were identified by searching 2014 MeSH. (https://www.nlm.nih.gov/mesh/meshhome.html#, accessed on Oct.30, 2014). “Myocardial Ischemia” was determined to be an appropriate MeSH term to search PumMed. Therefore, the search strategy “Myocardial Ischemia and Genetic” was applied in PubMed to identify all related published studies that investigated relationships between IHD and human genes. In addition, we used the names (strings) of all human genes from NCBI to select more specific literatures. We obtained a total of 15,670 records covering 1,723 human genes. There were 442 overlapping genes between the 604 IHD-associated genes from the CADgene database and 1,723 genes in the literatures. After manually checking the remaining 1,281 genes in PubMed, we identified 450 IHD-associated genes (see Table S2) and excluded 831 genes (see Table S3). Among the 831 genes, the string names of 36 genes (36/831 = 0.0433 < 0.05, potentially false negatives) were common and likely referred to other things. For example, DBP often referred to diastolic blood pressure and not a gene name, and therefore, hits with DBP were directly not regarded as associated genes. A total of 383 IHD-associated genes appeared in the OMIM database and GWAS after using corresponding disease names in Table 1 as keywords to search the associated genes in Disease-Connect (Liu et al., 2014). We identified 381 genes that overlapped with the above results. Finally, we obtained 1,056 IHD-associated genes (see Table S4) which mainly were relevant to the molecular functions of response to wounding (GO:0009611) and inflammatory response (GO:0006954) et al, and the enriched pathways, such as complement and coagulation cascades(hsa04610) and cytokine-cytokine receptor interaction (hsa04060) (see Table 2 for top 10 significant GO terms and enriched pathways).

**Table 1:** MeSH term headings and their corresponding disease names in the context of ischemic heart disease.

**Unique MeSH term ID**	**MeSH headings**	**Corresponding disease names**
D054058	Acute Coronary Syndrome	Acute Coronary Syndrome
D000787	Angina Pectoris	Angina Pectoris
D000788	Angina Pectoris, Variant	Variant Angina Pectoris
D060050	Angina, Stable	Stable Angina
D000789	Angina, Unstable	Unstable Angina
D003324	Coronary Artery Disease	Coronary Artery Disease
D017566	Microvascular Angina	Microvascular Angina
D056988	Anterior Wall Myocardial Infarction	Anterior Wall Myocardial Infarction
D056989	Inferior Wall Myocardial Infarction	Inferior Wall Myocardial Infarction
D009203	Myocardial Infarction	Myocardial Infarction
D017202	Myocardial Ischemia	Myocardial Ischemia
D015428	Myocardial Reperfusion Injury	Myocardial Reperfusion Injury
D017682	Myocardial Stunning	Myocardial Stunning
D012770	Shock, Cardiogenic	Cardiogenic Shock
D016757	Death, Sudden, Cardiac	Cardiac Sudden Death

**Table 2:** Top 10 Significant GO terms and pathways (CPV < 0.01) in the ischemic heart disease associated gene set.

**GO terms**	**CPV**	**Pathway**	**CPV**
Response to wounding(GO:0009611)	6.78E-28	Complement and coagulation cascades(hsa04610)	2.09E-7
Inflammatory response(GO:0006954)	1.20E-27	Cytokine-cytokine receptor interaction(hsa04060)	5.55E-6
Circulatory system process(GO:0003013)	3.25E-25	Malaria(hsa05144)	1.02E-5
Blood circulation(GO:0008015)	3.86E-25	NF-kappa B signaling pathway(hsa04064)	4.03E-5
Regulation of response to wounding(GO:1903034)	5.20E-25	Inflammatory bowel disease (IBD)(hsa05321)	5.21E-5
Response to oxygen-containing compound(GO:1901700)	5.20E-25	TNF signaling pathway(hsa04668)	6.32E-5
Response to lipid(GO:0033993)	2.09E-24	Pathways in cancer(hsa05200)	2.51E-4
Response to lipopolysaccharide(GO:0032496)	1.82E-22	Chagas disease (American) (trypanosomiasis)(hsa05142)	3.90E-4
Response to molecule of bacterial origin(GO:0002237)	3.10E-22	Legionellosis(hsa05134)	4.29E-4
Regulation of response to external stimulus(GO:0032101)	4.43E-22	Leishmaniasis(hsa05140)	7.69E-4

### IHD disease modules

From the PPI network derived from String V9.1 database, we obtained 157 potential disease modules covering a total of 11,380 proteins. As shown in Table S5, there were 997 IHD-associated genes appearing in 157 potential disease modules. Thus, the average proportion of IHD-associated genes appearing in potential disease modules was 8.76% (= 997/11,380). Using the relative risk measurement, we finally identified 8 disease modules (i.e., Modules 195, 204, 95, 203, 194, 212, 59, and 146) with the proportion of associated genes in corresponding disease modules >8.76% × 3 = 26.28%, see Table 3).We also analyzed the enrichment GO and pathway of disease modules and These disease modules had enriched pathways on Renin-angiotensin system (hsa04614), Vitamin digestion and absorption (hsa04977) et al, with the molecular functions of angiotensin maturation(GO:0002003), extracellular space(GO:0005615) et al (see Table 4 for the top significant GO terms and pathway in disease modules).

**Table 3:** Ischemic heart disease associated genes, symptom-related genes, known classical formula-related targets and IHD-related targets appearing in disease modules.

**Module**	**IHD-associated genes**		**Symptom-related genes**		**Known classical formula-related and IHD-related targets**		
	**No. IHD-associated genes/genes in the modules**	*p*-value^*^	**Symptoms genes/genes in the modules**	***p*-value^Δ^**	**Known IHD-related targets**	**Known classical formula-related targets for BS with IHD**	**Known classical formula-related targets for PS with IHD**
Module 195	10/24	2.55E-4	10/24	1.08E-4	4	3	1
Module 204	12/31	1.11E-4	5/31	0.183	1	1	0
Module 95	76/204	1.45E-29	40/204	1.83E-7	7	19	15
Module 203	21/59	1.17E-6	10/59	0.033	4	0	0
Module 194	22/62	6.57E-7	10/62	0.040	7	3	0
Module 212	12/37	4.23E-4	8/37	0.015	0	1	2
Module 59	23/77	4.21E-6	23/77	0.037	6	2	2
Module 146	44/149	2.21E-13	16/149	0.227	9	2	6

(1) P value* represents the p value of the proportion of IHD-associated genes in the modules compared to the average distribution of 997 IHD-associated genes in 157 potential disease modules. (2) P value^Δ^ represents the p value of the proportion of PSCS symptom-related genes in 157 potential disease modules compared to the average distribution of 890 symptom-related genes. (3) The color rows represent the modules included significantly more symptom-related genes were designated PSCS syndrome modules.

**Table 4:** The top significant GO terms and pathways in disease modules.

**Module**	**GO enrichment function analysis**		**Pathway enrichment function analysis**	
	**GO terms**	**CPV**	**Pathways**	**CPV**
Module 195	Angiotensin maturation(GO:0002003)	7.25E-10	Renin-angiotensin system(hsa04614)	2.91E-10
Module 204	Extracellular space(GO:0005615)	2.05E-6	Vitamin digestion and absorption(hsa04977)	0.039
Module 95	Response to bacterium(GO:0009617)	4.39E-21	Toll-like receptor signaling pathway(hsa04620)	1.88E-9
Module 203	Extracellular space(GO:0005615)	1.08E-16	Complement and coagulation cascades(hsa04610)	5.59E-7
Module 194	Blood coagulation(GO:0007596)	2.79E-23	Complement and coagulation cascades(hsa04610)	1.23E-32
Module 212	Platelet alpha granule lumen(GO:0031093)	3.59E-27	TGF-beta signaling pathway(hsa04350)	1.96E-3
Module 59	Cellular lipid metabolic process (GO:0044255)	9.59E-42	Ether lipid metabolism(hsa00565)	4.42E-38
Module 146	G-protein coupled receptor signaling pathway(GO:0007186)	1.51E-58	Neuroactive ligand-receptor interaction(hsa04080)	1.02E-40

### PSCS syndrome modules

In the Human Symptoms Disease Network (Zhou et al., 2014b), we identified 1,219 genes corresponding to the following seven symptoms: ecchymosis (MeSH ID: D004438), body weight (MeSH ID: D012816), obesity, morbid (MeSH ID: D009767), cyanosis (MeSH ID:D003490), lethargy (MeSH ID: D053609), and chest pain (MeSH ID:D002637) (see Table S6).We mapped a total of 890 symptom-related genes into 157 potential disease modules. Compared to the average proportion (890/11,380 = 7.82%), Modules 95 and 195 (as shown in Figure 2C), which included significantly more symptom-related genes (see Table 3), were designated syndrome modules.

### Validation

A total of 133 distinct known classical formula-related targets for PS with IHD (see Table S7) and 152 distinct classical formula-related targets for BS with IHD (see Table S8) from HIT were mapped into disease modules. There were 3 BS-related targets and 1 PS-related target in Module 195 and 19 BS-related targets and 15 PS-related targets in Module 95 (as shown in Figure 2C), suggesting these two modules represent PSCS with IHD based on the co-occurrence of prescriptions and syndromes (Deng et al., 2012; Lu et al., 2014).

Overall, we obtained 158 distinct IHD-related targets, in which 34 distinct IHD-related targets were curated from the TTD database (47 targets in total, see Table S9) and 140 distinct IHD-related targets from the DrugBank database (259 targets in total, see Table S10). When these 158 targets were mapped into disease modules (see Table S5), as shown in Figure 2C, Module 195 contained 4 IHD-related targets and Module 95 contained 7 IHD-related targets (as shown in Figure 2C). This directly demonstrated the reliability of Modules195 and 95 from the pharmacological perspective.

We identified corresponding symptom-gene relationships for 3 symptoms in Module 95 in the HPO (as shown in Figure 2C): obesity (HP:0001513) -WWOX (Gene ID:51741), cyanosis (HP:0000961)-GJA1(Gene ID:2697), and chest pain(HP:0100749)-WWOX(Gene ID: 51741). However, we found no corresponding symptom-gene relationships for the symptom-related genes in Module 195.

Based on the literatures, matrix metallo peptidase 9 (MMP9) in Module 95, which was simultaneously an IHD-associated gene/IHD-related target/classical formula-related target for BS and PS with IHD, was chose as an example for analysis. A synthesis of available evidence suggested that MMP9–1562C/T polymorphism was a risk factor for CHD (Liu et al., 2013), and MMP9 serum levels were consistently associated with markers of carotid atherosclerosis and lesion vulnerability (Blankenberg et al., 2003; Tanner et al., 2011; Silvello et al., 2014). Importantly, two studies in China demonstrated that MMP9 levels in the blood decreased significantly after treatment with *Shan Zha Xiao Zhi Decoction* (Li, 2011; Wang et al., 2012b) or *Danlou Tablet* (Wang et al., 2012a) (another two formulas use to treat PSCS with IHD) compared with the levels prior to treatment (*p* < 0.01). Additionally, two genes in Module 195, specifically angiotensin-converting enzyme (ACE) and plasminogen activator, urokinase (PLAU), were IHD-associated genes/known IHD-related targets / symptom-related genes. ACE was the target of inhibitor drugs, such as ramipril, trandolapril, and benazepril, all FDA-approved, which were used to treat hypertension to reduce the rate of death, myocardial infarction, and stroke in individuals at high risk for cardiovascular events. Our study indicated that ACE was related to the symptom morbid obesity. According to Edson Lucas Santos's research, the ACE inhibitor enalapril decreased body weight gain and increased life span by activating PPARγ in adipose tissue (Santos et al., 2009). Additionally, based on a recent clinical trial, PLAU appeared to be related to the symptom chest pain. Furthermore, soluble urokinase plasminogen activator receptor was a strong predictor of adverse long-term outcomes and improves risk stratification beyond traditional risk variables in chest pain patients admitted with suspected non-ST-segment elevation acute coronary syndrome (Lyngbaek et al., 2013). *Bao Xin decoction* (another formula to treat PSCS with IHD) was effective in curing CAD with stable angina pectoris and acting by inhibiting serum interleukin 6 (IL-6), intercellular adhesion molecule 1 (ICAM-1) and tumor necrosis factor-α (TNF-α) levels and decreasing inflammatory reactions (Peng et al., 2011). Notably, IL-6 and ICAM-1 appeared in Module 95. Another study reported significantly increased (*p* < 0.05) serum ICAM-1 levels in acute coronary syndrome patients, and serum ICAM-1 levels were higher in patients with PSCS (Ma et al., 2017).

Finally, evidence suggested that corresponding targets in Module 195 may be used to treat other types of disease for which PSCS was the main syndrome or protein levels correlated with PSCS. For example, C-reactive protein (CRP) mRNA was the target of ISIS-CRPRx, which was used to treat trial fibrillation (Adis Insight, 2017). According to another study, BS and PS represented the primary pathogenesis of trial fibrillation with CHD based on the distribution of TCM syndrome in 259 patients with a trial fibrillation and CHD (Yin et al., 2007). Nitric oxide synthase 2 (NOS2), which also appeared in Module 195, was the target of triflusal, which was used to prevent cardiovascular events such as stroke. A clinical epidemiological survey of 1,418 stroke patients showed BS and PS were both the main pathological factors of apoplexy throughout stroke: during the acute stage, the rate of PSCS occurrence was 66.4%. During the recovery period, the rate of PSCS was 68.7%. In the sequelae phase, the rate was 61.2% (Yang et al., 2004).

## Discussion

Based on our study, Modules 195 and 95 may be closely associated with PSCS with IHD. From the enrichment function analysis results of these two potentially syndrome modules, we also further validated the relativity of the two modules with PSCS with IHD. For example, it showed that MyD88-dependent pathway that leads to the production of proinflammatory cytokines, such as TNF-α, interleukin-6 (IL-6), interleukin-8 (IL-8), and interleukin-1β(IL-1β), accompanied by the rapid activation of NF-kappa B and MAPK signaling pathways (KEGG)^1^ (as shown in the website Toll-like receptor signaling pathway, 2016, red objects indicated proteins that appear in Module 95). IL-1β, TNF-α, and IL-6 stimulated the liver to produce high-sensitivity CRP (hs-CRP) (Yamashita et al., 2015), which had a strong relationship with the recurrent events of cardiovascular diseases as shown in several randomized clinical trials (Ridker et al., 2011; Everett et al., 2013). One study reported that was fully compatible with the existence of a multi-cytokine resistin pathway in cells and tissues (resistin affects IL-1β, IL-6, IL-8, IL-12, and TNF-α expression, thus suggesting the existence of a multi-cytokine “resistin pathway”). Additionally, another study demonstrated that *Danlou tablets* [the only formula approved by the China Food and Drug Administration to treat PSCS with IHD (Yang and Wang, 2012)] reduced levels of serum resistin, endothelin-1 (ET-1), IL-6 and TNF-α, improved NO levels and relieved vascular endothelial injury in atherosclerotic model rats (Miu et al., 2016).

In our study, a total of 1,056 IHD-associated genes were curated after validating each disease-gene relationship in the biomedical literatures. This gene list may be the most comprehensive phenotype-genotype association data repository for IHD molecular mechanisms. According to our functional enrichment analysis results for the IHD-associated gene set (see Table S11), the primary pathology consisted of response to wounding, inflammatory response, circulatory system process, blood circulation, regulation of response to wounding, response to oxygen-containing compound, response to lipid, response to lipopolys accharide, response to molecule of bacterial origin, and regulation of response to external stimulus. IHD was a complex disease, and there had been many hypotheses to explain its pathology and mechanism, such as the thrombosis theory (Zaman et al., 2000), lipid infiltration (Castelli et al., 1986), response to injury hypothesis, oxidative hypothesis (Stocker and Keaney, 2004), immune and inflammatory mechanisms (Epstein and Ross, 1999), shear stress hypothesis (Ku et al., 1985), and others. The function analysis results for the IHD-associated gene set were mostly consistent with current knowledge and provided strong evidence that the genes we integrated in the study were reliably related to IHD. However, reasons why the results were not completely consistent with present knowledge of IHD may include: (1) interactome incompleteness: current PPI maps had only covered < 10% of all potential interactions (Hart et al., 2006; Venkatesan et al., 2009; Vidal et al., 2011). Thus, many additional isolated proteins may be part of a single disease module, but the missing links had not been isolated. (2) False positives: not all genes in the seed gene set had a mechanistic association with IHD.

Unlike the understanding of a syndrome in the context of the neuro-endocrine-immune network (Li et al., 2007, 2013), we proposed a network medicine module-based strategy by integrating symptoms-gene relationship, then validating by compound-target-disease associations and known knowledge. And we identified relevant modules representing one syndrome of a complex disease under the guideline of integrating pattern classification and biomedical diagnosis using a systems biology approach (Lu et al., 2012). The relationships between diseases and syndromes were complex, one disease may express different syndromes, while one syndrome may appear in different diseases. For example, one previous study had reported that 223 patients with acute cerebral infarction could be divided into six syndromes: wind syndrome, fire syndrome, PS, BS, deficiency of qi syndrome, and yin deficiency causing hyperactivity of yang syndrome (Li et al., 2011). To identify the modules representing PSCS with IHD as accurately as possible, we first collected PSCS symptom-related genes to represent a syndrome where “syndrome” consisted of the overall “manifestation” of human body pathological and physiological changes expressed in the form of information obtained from four diagnostic methods: inspection, listening and smelling, interrogation, and pulse feeling and palpation (Shi et al., 2014a). To confirm the reliability of the PSCS modules, network medicine approaches were implemented to integrate the data from multiple databases in which network pharmacology approaches provided new insight to understand TCM syndrome from a scientific perspective (Li et al., 2013; Hao da and Xiao, 2014; Fang et al., 2017). Besides, classical formula-related target information was integrated to demonstrate that Modules 195 and 95 representing PSCS with IHD from the classical formula perspective. Additionally, because known classical formula-related targets are potentially less credible than known IHD-related targets, IHD-related targets were used for further confirmation. Finally, the HPO and published studies were used to validate our results. After collecting different types of evidence as completely as we could, our study suggested that these two modules represent PSCS with IHD.

The genes and proteins that appeared in Module 195 and Module 95 may be biomarkers for PSCS with IHD, and these modules also comprise classical formula-related target and IHD-related target information, indicating the potential of these targets for the treatment of IHD and the discovery of new drugs based on Chinese formulae and herbs.

Our results were affected by incomplete data and may even be enriched for biased publications. Furthermore, different algorithms may result in different division results for the topological modules. Due to its timing and conditions, this study did not experimentally validate the two syndrome modules. However, researchers had previously explored potential therapeutic targets by comparing the target proteins of classic traditional TCM herbal formulas and modern drugs used to treat other types of diseases, as described in Qianru Zhang's study (Zhang et al., 2017).

Notwithstanding these challenges and shortcomings, we believed that this study puts forward a novel strategy to identify modules representing syndromes of complex diseases and provide insights into the molecular mechanism of PSCS with IHD. In future studies, we will incorporate experimental evidence and clinical research to validate the reliability of these two syndrome modules.

## Conclusion

We proposed a network medicine-based approach to identify the underlying molecular modules of PSCS complicated with IHD, which could be used for interpreting the pharmacological mechanisms of well-established Chinese herbal formulas (e.g., *Tao Hong Si Wu Decoction, Dan Shen Yin Decoction, Hunag Lian Wen Dan Decoction*, and *Gua Lou Xie Bai Ban Xia Decoction*). In addition, our results delivered novel understandings of the molecular network mechanisms of IHD phenotype subtypes with PSCS complications, which would be both insightful for IHD precision medicine and the integration of disease and TCM syndrome diagnoses.

## Author contributions

J-QH and X-ZZ: conceived and designed the research; W-MX, KY, L-JJ, J-QH, and X-ZZ: performed the following research projects: curation of the IHD-associated genes; W-MX, L-JJ, X-ZZ, KY, and J-QH: identification of disease and syndrome modules, enrichment function analysis; W-MX, KY, and X-ZZ: results validation; W-MX, KY, X-ZZ, and J-QH and W-MX: wrote the manuscript. All authors have reviewed and revised the manuscript.

### Conflict of interest statement

The authors declare that the research was conducted in the absence of any commercial or financial relationships that could be construed as a potential conflict of interest.

## Footnotes

^1^KEGG Toll-like Receptor Signaling Pathway - Homo sapiens (human) [Online]. KEGG. Available online at: http://www.genome.jp/kegg-bin/show_pathway?hsa04620/hsa:51311%09red/hsa:3569%09red/hsa:7100%09red/hsa:353376%09red/hsa:7096%09red/hsa:7097%09red/hsa:7124%09red/hsa:54472%09red/hsa:4615%09red/hsa:23643%09red/hsa:3576%09red/hsa:7099%09red/hsa:114609%09red/hsa:3553%09red/hsa:51284%09red/hsa:54106%09red/hsa:929%09red/hsa:51135%09red/hsa:3654%09red (Accessed May 5, 2017].

## Abbreviations

IHD: Ischemic heart disease
CAD: Coronary heart disease
TCM: Traditional Chinese Medicine
PS: Phlegm syndrome
BS: blood-stasis syndrome
PSCS: Phlegm-stasis cementation syndrome
NOS: nitric oxide synthase
NO: Nitric oxide
OMIM: Online Mendelian Inheritance in Man
GWAS: Genome-wide association study
HIT: Herb Ingredients' Targets
HPO: Human Phenotype Ontology
TTD: Therapeutic Target Database
ICD: International Classification of Diseases
GO: gene ontology
PPI: Protein-protein interaction
HPO: Human Phenotype Ontology
HSDN: Human Symptoms-Disease Network
KEGG: Kyoto Encyclopedia of Genes and Genomes
CPV: Corrected p-values
MMP9: Matrix metallo peptidase 9
ACE: Angiotensin-converting enzyme
PLAU: Plasminogen activator, urokinase
IL-6: Interleukin 6IL-8, Interleukin-8
IL-8: Interleukin-8
ICAM-1: Intercellular adhesion molecule 1
TNF-α: Tumor necrosis factor-α
CRP: C-reactive protein
MAPK: Mitogen-activated protein kinase
NCBI: National Center for Biotechnology Information.
